# End-of-life care for people with dementia on a green care farm during the COVID-19 pandemic: a qualitative study

**DOI:** 10.1186/s12877-022-03584-5

**Published:** 2022-12-12

**Authors:** Kirsten D. Smit, Sascha R. Bolt, Bram de Boer, Hilde Verbeek, Judith M. M. Meijers

**Affiliations:** 1 Utrecht University, Nursing Sciences, program in Clinical Health Sciences, University Medical Center Utrecht, Utrecht, The Netherlands; 2 Maastricht University, Care and Public Health Research Institute, Department of Health Services Research, Maastricht, The Netherlands; 3 Living Lab in Ageing and Long-Term Care, Maastricht, The Netherlands; 4 Tranzo, Tilburg School of Social and Behavioral Sciences, Tilburg University, Tilburg, Netherlands; 5 Zuyderland Care, Zuyderland Medical Center, Dr. H. van der Hoffplein 1, 6162 BG Sittard-Geleen, The Netherlands

**Keywords:** Dementia, Palliative care, Green care farms, Experiences of healthcare workers and family caregivers, COVID-19

## Abstract

**Objective:**

Green care farms combine agriculture production with health-related, social and educational services. In the Netherlands, they form an alternative to traditional nursing homes for people with dementia. Green care farms that offer 24-hour care, also offers end-of-life care. To date, little is known about end-of-life care for people with dementia on green care farms. This study aimed to explore the experiences of healthcare workers and family caregivers with end-of-life care for people with dementia who died on a green care farm.

**Design:**

An explorative, descriptive qualitative design with a phenomenological approach.

**Setting and participants:**

A purposive sample of 15 participants – seven healthcare workers and eight family caregivers - from three green care farms in the Netherlands.

**Methods:**

Semi-structured, in-depth interviews were conducted to explore participants’ experiences with end-of-life care, including topics such as advance care planning, the influence of COVID-19, and bereavement support. Transcripts were thematically analysed using Braun and Clarke’s approach.

**Results:**

Four main themes were extracted: 1) tailored care and attention for the individual resident, 2) reciprocal care relationships between healthcare workers and family caregivers, 3) compassionate care and support in the dying phase, and 4) the influence of COVID-19 on end-of-life care.

**Conclusion and implications:**

The overall experience of the healthcare workers and family caregivers was that end-of-life care offered on green care farms is person-centred and compassionate and is tailored to the person with dementia and their family caregivers. Despite the COVID-19 pandemic, healthcare workers and family caregivers were satisfied with end-of-life care on the green care farms. Green care farms may offer a valuable alternative care setting for people with dementia in their last phase of life. More research is needed to investigate green care farms’benefits compared to other, more traditional settings.

## What is already known?

- Previous studies have already investigated about the end-of-life care for people with dementia in nursing homes.
- Previous studies indicated that they were positive about life on a care farm for people with dementia.

## What this paper adds?

- This study demonstrates that end-of-life care for people with dementia on a green care farm is experienced as person-centred and tailored care.
- Relationships between healthcare workers and family caregivers on green care farms are intensive and involve reciprocal care and support.
- The person with dementia is central during the end-of-life care on green care farms.

## Background

In 2018, around 80,000 people with dementia were living in nursing home settings [1]. In the Netherlands, different types of nursing homes exist. One innovative form of long-term care is a green care farm that provides 24-hour care [2]. A green care farm differs from a regular nursing home in that it combines living environments and agricultural production with health-related, social and educational services [2]. Green care farms are an alternative form of traditional nursing homes; the residents of the green care farms are more active, spend more time outdoor, have more social interactions and have a higher quality of life [3]. Living on a green care farm has a positive effect on resident outcomes such as self-worth and a sense of autonomy and identity [2, 4].

Earlier research indicated that 70% of people with dementia die in a nursing home setting [5]. Green care farms may offer a home for life and thus also offer end-of-life care. During end-of-life care in a regular nursing home, the family caregivers and healthcare workers have an important role. Family caregivers report more positive experiences with green care farms compared to traditional nursing homes [6]. Clear and adequate communication between healthcare workers and family caregivers is essential at the end of life of persons with dementia [7]. Family caregivers’ reports show that humane and compassionate care and attention from healthcare workers towards the resident and family can facilitate the memory of a peaceful death for their loved one with dementia [8]. Family caregivers may also experience distress at the end of life of a loved one with dementia, and need support [9].

The end-of-life care for people with dementia is complex because each person is different and a wide range of potential problems exist, both physical and mental, along with cognitive challenges [10]. At the end of life and in advanced stages of dementia, the focus on optimal comfort is important in the care for persons with dementia and their families [11, 12].

There is limited research about end-of-life care provided on green care farms [13]. It is currently unknown how family caregivers and healthcare workers experience the provision of end-of-life care for people with dementia on green care farms. In end-of-life care for people with dementia, family caregivers and healthcare workers have an important role. Due to the cognitive decline in persons with dementia, family caregivers are often advocates for their relatives [7, 8].

The research question of this study is: what are the experiences of healthcare workers and family caregivers with end-of-life care for people with dementia on green care farms with 24-hour care?

This study was conducted during the COVID-19 pandemic, which impacted end-of-life care for residents, and the experiences of family caregivers and healthcare workers in long-term care, due to measures such as social distancing and visiting restrictions [14]. Therefore, we additionally explored the influence of the COVID-19 pandemic.

## Methods

### Study design

The research uses an explorative, descriptive and qualitative design with a phenomenological approach [15, 16].

### Study setting and sampling

The study was carried out in green care farms that provide 24-hour care for people with dementia in the Netherlands. Participants were healthcare workers at the green care farms and family caregivers of residents with dementia. Table 1 presents the inclusion criteria for participation in the study.

**Table 1:** Inclusion criteria

Healthcare workers are eligible to participate if they meet the following criteria:	The family caregivers are eligible to participate if they meet the following criteria
1. first responsible healthcare worker of the person with dementia	1. First contact person of the person with dementia
2. was present in the green care farm with 24 hours care during the end-of-life care	2. their relative has the diagnose dementia
3. is working at the green care farm for at least one year	3. their relative with dementia has been living at the farm for at least three months
4. have sufficient proficiency in the Dutch language	4. their relative with dementia died in the green care farm
5. gave informed consent.	5. have sufficient proficiency in the Dutch language
	6. gave informed consent

We recruited a purposive sample of participants [17]. The researchers approached managers of six green care farms. The managers contacted the healthcare workers and the family caregivers. Six interviews took place online, three interviews at the persons home and six interviews at the green care farms. One family caregiver declined participation, because the topic was too sensitive.

All participants gave written informed consent. The study was approved by the Medical Ethics Committee of Zuyderland. The study was part of a project entitled DEDICATED. The study was conducted in line with the Declaration of Helsinki (2013).

### Data collection

Data were collected in March and April 2021, using semi-structured interviews. Interview topics were based on existing literature concerning end-of-life care for people with dementia and on the End-of-Life in Dementia (EOLD) instruments (Table 2) [13, 18].

**Table 2:** Topics for the interview

Healthcare worker	Family caregiver
General policy about palliative care on the green care farm and policy with Covid-19.	Brief description of the person with dementia (case)
Brief description of the person with dementia (case)	Experiences with care at the end of life
End of life	Policy focuses on the case
Care provision and end-of-life policy on the care farm.	The communication with family caregivers from the healthcare worker around the end of life
Communication with healthcare worker around the end of life	Support
Communication with the person with end-of-life dementia	
Support of colleagues	
First question: Can you briefly describe what palliative care is on a green care farm?	First question: Can you give me a brief description of what the person with dementia was on the green care farm?’

The participants were asked to provide demographic information on their age, gender, work experience, level of education and relationship to the person with dementia. All the interviews were audio-recorded and transcribed verbatim. A summary of the transcript was sent to the participants for approval. The data collection continued until reaching data saturation.

### Data analysis

The thematic analysis method by Braun and Clarke was used to analyse the interview transcripts [18]. NVivo 12 software was used to perform the analysis. The analysis consisted of six steps to identify recurring themes from the interviews.

In phase one, familiarisation with the data, the main researcher transcribed and read the interviews. The first notable points from the interviews were discussed with the research team. In the second phase, generating initial codes, two researchers separately coded four interviews. Afterwards, they got together to reach a consensus about the coding scheme. In phase three, searching for themes, the codes were categorised and categories were collapsed into themes. Two researchers reached a consensus on the themes, which were described separately for healthcare workers and family caregivers. In phase four, reviewing the themes, the main researcher drafted a thematic map, to check the correspondence between the themes and the codes. Following this, the themes, along with any discrepancies, were discussed with the research group to reach a consensus. In phase five, defining and naming themes, the research group defined the main themes using the categories and codes. In phase six, producing the report, the themes were linked to the research question and described in detail.

## Results

Three of the approached green care farms had eligible participants. These three green care farms housed, respectively, 14, 27 and 60 residents. Two of the green care farms consist of multiple farmhouses. All three have large gardens with animals and shared living rooms with daytime activities.

Seven healthcare workers and eight family caregivers agreed to participate. Table 3 presents the demographic characteristics of the 15 participants.

**Table 3:** Case descriptions

**People with dementia**						
**Pseudonym**	**Sex**	**Age in years at death**	**Connection to the family caregiver**		**Length of stay on the green care farm**	**Character**
Alex	Male	95	Partner		4 years	Loved to paint and was very accessible. Has lived on a farm. Died on Covid-19.
Noor	Female	79	Partner		2 years	Sweet and difficult to deal with. Died on Covid-19.
Hans	Male	80	Father		Few months	Loved to play games, but slept a lot. Died on Covid-19.
Anna	Female	78	Mother		5,5 years	Sweet and caring, happy to helped. Slept a lot.
Paul	Male	94	Father		2 years	Social man, loved to walk outside.
Joy	Female	92	Mother		10 months	Sweet, cheerful and caring, happy to helped. Has lived on a farm. Survived Covid-19, but broke her hip.
Jan	Male	89	Partner		Few months	Lovely man, liked to draw.
Ted	Male	84	Partner		5 months	Liked to draw and to look outside the window to the garden.
**Healthcare workers**						
**Pseudonym**	**Sex**	**Age in years**	**Working in healthcare**	**Level**	**Working on the green care farm**	**Additional information**
Eva	Female	27	11 years	4	3 years	Gerontologist & manager day care.
Sara	Female	56	38 years	5	10 years	Knowledge of mental health care. Palliative care expert. Manager care.
Johan	Male	57	40 years	4	15 years	Green care farm manager.
Ella	Female	56	30 years	5	6 years	Palliative care expert. Manager care.
Kelly	Female	57	36 years	3	4 years	Different experiences in nursing care homes.
Sanne	Female	50	2 years	3	2 years	Recently graduated, actively developing palliative care.
Suus	Female	54	2 years	3	2 years	Recently graduated, she is passionate about palliative care.

**Fig. 1:**
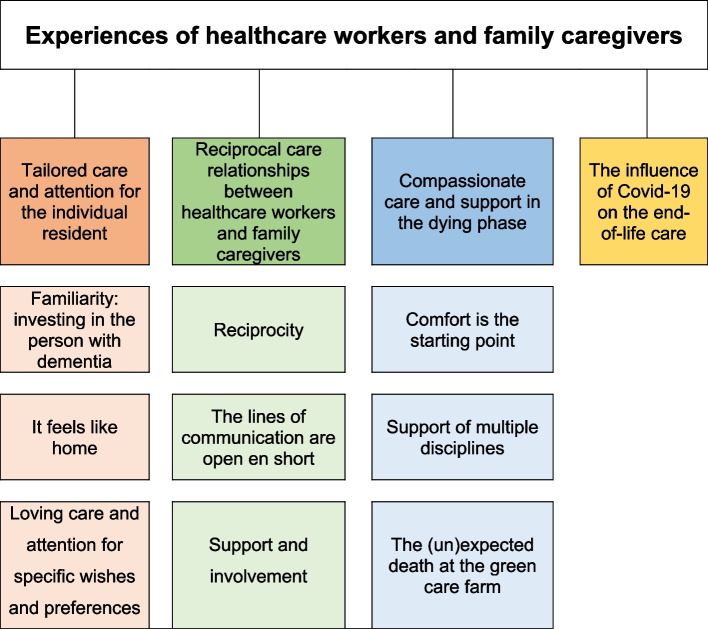
Themes and subthemes from the analysis

The thematic analysis of the interviews with healthcare workers and family caregivers resulted in four main themes: 1) tailored care and attention for the individual resident, 2) reciprocal care relationships between healthcare workers and family caregivers, 3) compassionate care and support in the dying phase and 4) the influence of COVID-19 on end-of-life care (Fig. 1).

### Tailored care and attention for the individual resident

#### Familiarity: investing in the person with dementia

All healthcare workers and family caregivers indicated that the resident’s individual needs are the primary focus within the green care farm. The healthcare workers indicated that it is important to get to know the resident well and to learn about their life stories to make an adequate inventory of the person’s needs.

The family caregivers indicated that they noticed this tailored care approach especially through small things. For instance, the residents were called by their first names by the healthcare workers and tailored activities were offered for the residents, such as painting. The healthcare workers also took sufficient time for the residents and they knew what was important to them.

The healthcare workers felt that they were able to take more time with the residents on the green care farm, compared to other settings*. “I worked in a nursing home for a while, the people were all nice there, but here I can take the time for them. I know them.” (Sanne, healthcare worker).*

#### It feels like home

For the family caregivers, the ambiance of the green care farms made it feel like home. Any requests that they had were listened and adhered to. Healthcare workers indicated that tailored care is the central focus, especially during the dying phase.

Family caregivers felt grateful that their relative spent the final phase of life on the green care farm, where they could still feel ‘at home’. “*So what I think is very important about life in such a care farm, after leaving home, is that they try to stimulate the ambiance of home as much as possible.” (Partner of Alex, family caregiver).*

#### Loving care and attention to specific wishes and preferences

The healthcare workers indicated that they knew the residents well, which helped them to be aware of personal needs and preferences in the dying phase. For example, they would sometimes invite a pastor, play a resident’s favourite music or make food that is loved by the person. In addition, special wake baskets are available during the dying phase. These contain information for the family caregivers and special fragrance oils and creams for the resident to enhance comfort. *“We take the person by the hand until the end.” (Ella, healthcare worker).* Family caregivers noted and appreciated the attention to residents’ specific preferences and the opportunities to experience moments of joy even in the dying phase.

### Reciprocal care relationships between healthcare workers and family caregivers

#### Reciprocity

The family caregivers were actively involved in the care of their relative from admission until the end of life at the green care farm. During the residents’ personal care activities, family caregivers were welcome to participate. They were frequently asked about their ideas and opinion about the care provided to their relatives. Reciprocity was evident for both the healthcare workers and family caregivers. Both parties considered their efforts as teamwork to optimise care for the person with dementia.

The relationship between the healthcare workers and the person with dementia was often very strong as well. For example some of the healthcare workers had attended former residents’ funerals. In turn, family caregivers appreciated this devotion.

Family involvement was also substantial in the end-of-life phase. For instance, they were generally allowed to stay at the farm and sleep over whenever they wanted. Special rooms were available for family caregivers to stay the night, or an extra bed would be placed in their relative’s room.

“*Yes all courtesies. And also sleeping there, everything was fine. I put a mattress on the floor.” (Daughter of Anna, family caregiver).*

#### The lines of communication are open and short

The participants experienced their mutual communication as positive and the communication lines between the healthcare workers and family caregivers were short. The contact was through regular calls and text messages with photos as well as through in-person visits. The family caregivers were often frequently present during the dying phase.

“*I could always text the manager. Or call if we had something. There were newsletters. … But I must say that the nurse guided us well, explained things and reassured us.” (Daughter of Hans, family caregiver).*

Healthcare workers indicated that family caregivers must be well informed about the care that is being provided, especially with palliative sedation. Family caregivers were included in every decision that had to be made during the dying phase. After the death of the resident, informal contact was maintained. For example, some family caregivers came back to the green care farm to work as volunteers.

#### Support and involvement

The healthcare workers explained that they pay attention to the family caregivers’ feelings, which was appreciated by the family caregivers. Even before the dying phase begins, regular conversations take place between them about future care scenarios and to answer any questions, which promotes an effective and trusted care relationship. The family caregivers indicated that the involvement of the healthcare workers was extensive in the dying phase. “*I went back to visit and cut out two paper hearts, and stuck them on the window ... And then a very sweet care worker came in, and he says shall I take a picture? Then he filmed our last contact.” (Partner of Alex, family caregiver).* In addition, after a resident has passed away, the healthcare workers have a special way of saying goodbye utilising an honorary hedge of the residents and healthcare workers.

### Compassionate care and support in the dying phase

#### Comfort is the starting point

From the perspective of all the healthcare workers, comfort was the primary focus of care in the dying phase. They emphasised the need for compassionate care, to enhance the residents’ comfort in their last week or days before death.

They saw palliative sedation as a means to prevent burdensome symptoms such as pain and anxiety, which supported a peaceful death. “*Of course we always say, you don’t have to be in pain.” (Johan, healthcare worker).*

The family caregivers were satisfied because they felt that their relative had died peacefully. The focus on comfort helped the family caregivers to feel comfortable as well as to accept their relative’s death.

#### Support of multiple disciplines

The participants indicated that during the dying phase, a physician, psychologist, nurses and nurse assistants were involved. The physician determined the policy together with the family and indicated when it was the right time to initiate palliative sedation. The healthcare workers stated they have close contact with the physician, which ensures clarity about care and treatment plans, which means that action can be taken quickly. “W*e try to keep it as comfortable as possible, and if you see that it is not the case, we will consult the physician. Fortunately, those are short communication lines.” (Sanne, healthcare worker)*.

In addition, healthcare workers indicated that a psychologist can play a valuable role by explaining the residents behaviour during the dying phase.

The healthcare workers and family caregivers confirmed that knowledge about end-of-life care varies within the team of healthcare workers. Nurses in particular have valuable knowledge, but there are also nurse assistants who have extensive knowledge and experience. The healthcare workers suggested that it is helpful to work with a shared guideline that all the healthcare workers, from different disciplines, can refer to.

#### The (un)expected deaths at the green care farm

The healthcare workers indicated that it varies whether the passing away of a resident was expected or not. Some deaths are very sudden and caught healthcare workers by surprise. Some residents had been bedridden for as little as 2 days before passing away. For most of the family caregivers, it felt as though the dying process occurred very quickly.

The healthcare workers indicated that the dying phase is generally short on the green care farm. According to some of the healthcare workers, the environment of the green care farm encourages activity, which may be a reason for the short time spent in bed before death. “*I have always held the opinion that people who spend a lot of time in the open air, who are often stimulated, have long and useful days and feel valuable and worthy as human beings and experience a shorter dying phase.” (Ella, healthcare worker)*. The residents on the green care farms are encouraged to stay active during the day. They can engage in various activities, such as taking care of the animals, working in the garden, cooking food and joining individual or group activities in the living room.

### The influence of COVID-19 on the end-of-life care

During the COVID-19 pandemic, the green care farms had to adhere to specific national guidelines. Nonetheless, the healthcare workers indicated that the presence of family caregivers was necessary for saying goodbye properly and peacefully in the dying phase. “*Saying goodbye is only possible once.” (Sara, healthcare worker).*

The COVID-19 pandemic affected the attendance of family caregivers, but they were still allowed to visit at a distance with window visits, even if it concerned more than one family caregiver. Participants felt that personal protective equipment, such as masks and glasses, was extremely disadvantageous for the healthcare workers and family caregivers, as it hampered making contact with the person with dementia*.*

Some of the residents’ partners were also afraid of being infected with the virus themselves. Participants gave examples of the situation in which family caregivers were not allowed to a normal visit, but only pay a “window visit”, which had a major impact on the family caregivers. “*This had a great impact, as just before his death I was not allowed to visit for ten days, nobody could because there was a nurse who got COVID-19…I went back to him for the first time on Tuesday. For the first time in ten days.” (Partner of Jan)*.

## Discussion

This study explored the experiences of the healthcare workers and family caregivers with end-of-life care for people with dementia living on green care farms during the COVID-19 pandemic. The results showed that most experiences were characterised by personal attention for the resident and family caregivers, and tailored holistic care. This created a home-like ambiance on the green care farms. The reciprocal care relationship between the healthcare workers and family caregivers was also a positive experience, with the intensive involvement of both parties in the care for residents. During the dying phase, care is particularly compassionate, as comfort was considered essential for the person with dementia and family caregivers. The duration of the dying phase was usually short, and most residents remained active until their final days. This study was performed during the COVID-19 pandemic, reflecting some of the possible impacts of the pandemic on end-of-life care. For example, “window visits”, personal protective equipment and rapid deaths caused by COVID-19 were typical events during the pandemic.

The positive experiences with end-of-life care on green care farms, correspond with previous research that showed that family caregivers were positive about the overall personal care available on green care farms [6]. Healthcare workers in this study indicated that the comfort of the resident is essential during the dying phase, which has also been confirmed by previous research focusing on the regular nursing home setting [19]. Therein, comfort is described as the overarching goal of end-of-life care [19].

Previous research has suggested that family caregivers are more positive about the green care farm’s environment, activities and personal care than family caregivers in more traditional settings [6]. This is consistent with our study in which family caregivers praised the care environment. The active environment of the green care farms is stimulating for the residents, which corresponds with an earlier study in nursing homes that suggested that encouraging movement increases the residents’ quality of life [3, 20]. Our study participants suggest that the active environment of the green care farms could support the rather short dying phase of residents on green care farms.

Despite the COVID-19 measures, in this study, healthcare workers and family caregivers still experienced a sufficient level of contact and communication with each other. This finding contrasts with another study, which found that there was limited communication between healthcare workers and family caregivers during the COVID-19 period [21]. Moreover, research shows that the social support and rituals around death were limited for the dying phase during the COVID-19 pandemic [21]. Yet, the current study showed that the family caregivers felt much supported and that healthcare workers tried to carry out usual end-of-life rituals as much as possible.

A strength of this study is that it explored experiences from two perspectives: healthcare workers and family caregivers. Their experiences with end-of-life care involved similar themes. For this study, six green care farms were approached, but only three provided eligible participants who agreed to participate. This small number of farms represents a limitation, as the findings may not fully generalise to other green care farms. Not all the green care farms in and outside the Netherlands were included, which is a limitation because of potential selection bias.

For future research, it is recommended to study the effects of the green care environment on the length and trajectory of the end-of-life phase of people with dementia, compared to other small-scale living facilities, and traditional nursing homes.

## Conclusion and implications for practice

End-of-life care for people with dementia on a green care farm is experienced as person-centred and tailored care. Healthcare workers and family caregivers invest in an intensive, reciprocal care relationship. During the dying phase, the primary focus is on the comfort of the person with dementia. COVID-19 had possible impacts during the dying phase, however, end-of-life rituals were mostly continued. This study provides a starting point for doing future research on end-of-life care for people with dementia on a green care farm.

## Data Availability

All data generated/analysed are available upon reasonable request with corresponding author.
